# Plant and Bird Presence Strongly Influences the Microbial Communities in Soils of Admiralty Bay, Maritime Antarctica

**DOI:** 10.1371/journal.pone.0066109

**Published:** 2013-06-20

**Authors:** Lia C. R. S. Teixeira, Etienne Yeargeau, Fabiano C. Balieiro, Marisa C. Piccolo, Raquel S. Peixoto, Charles W. Greer, Alexandre S. Rosado

**Affiliations:** 1 Laboratório de Ecologia Microbiana Molecular, Instituto de Microbiologia Paulo de Góes, Universidade Federal do Rio de Janeiro, Rio de Janeiro, RJ, Brasil; 2 Biotechnology Research Institute, National Research Council of Canada, Montréal, QC, Canada; 3 Empresa Brasileira de Pesquisa Agropecuária (EMBRAPA) solos, Rio de Janeiro, RJ, Brasil; 4 Laboratório de Ciclagem de Nutrientes, Centro de Energia Nuclear na Agricultura, Universidade de São Paulo, SP, Brasil; University of Alberta, Canada

## Abstract

Understanding the environmental factors that shape microbial communities is crucial, especially in extreme environments, like Antarctica. Two main forces were reported to influence Antarctic soil microbes: birds and plants. Both birds and plants are currently undergoing relatively large changes in their distribution and abundance due to global warming. However, we need to clearly understand the relationship between plants, birds and soil microorganisms. We therefore collected rhizosphere and bulk soils from six different sampling sites subjected to different levels of bird influence and colonized by *Colobanthus quitensis* and *Deschampsia antarctica* in Admiralty Bay, King George Island, Maritime Antarctic. Microarray and qPCR assays targeting 16S rRNA genes of specific taxa were used to assess microbial community structure, composition and abundance and analyzed with a range of soil physico-chemical parameters. The results indicated significant rhizosphere effects in four out of the six sites, including areas with different levels of bird influence. *Acidobacteria* were significantly more abundant in soils with little bird influence (low nitrogen) and in bulk soil. In contrast, *Actinobacteria* were significantly more abundant in the rhizosphere of both plant species. At two of the sampling sites under strong bird influence (penguin colonies), *Firmicutes* were significantly more abundant in *D. antarctica* rhizosphere but not in *C. quitensis* rhizosphere. The *Firmicutes* were also positively and significantly correlated to the nitrogen concentrations in the soil. We conclude that the microbial communities in Antarctic soils are driven both by bird and plants, and that the effect is taxa-specific.

## Introduction

*Deschampsia antarctica* Desv. (*Poaceae*) and *Colobanthus quitensis* (Kunth) Bartl. (*Caryophyllaceae*) are the only two native vascular plants in Antarctica. Both species are widespread, usually growing together and well adapted for life in the coastal Maritime Antarctic [1]. A considerable increase in distribution and abundance of *D. antarctica* and *C. quitensis* in the western Antarctic has been reported and interpreted as a response to more favorable growing and reproductive conditions [2]. In some locations, the annual average temperature has increased by more than 1°C in the last 30–50 years [3]. In the Maritime Antarctic region, this increase in temperature has led to the thaw of glaciers, that exposed new areas of land [3], which could further increased the distribution of vascular plants [4].

Through root exudation, plants produce a variety of chemical compounds that may be used by soil bacteria for energy and biomass production [5]. Several authors have reported that this interaction selects for specific microbial communities in the rhizosphere, the portion of soil that is directly influenced by roots, producing the “rhizosphere effect” [6]–[8]. However, there is also strong evidence that soil type could have a more determinant role in shaping the microbial communities than the plant [9], [10]. Similarly, a recent study showed, using 16S rRNA gene pyrosequencing, that the microbial communities associated with the rhizospheres of *D. antarctica* and *C. quitensis* did not differ significantly [11]. However, this study did not compare bulk and rhizosphere soil, and it is thus still not known what level of influence vascular plants exert on Antarctic soil microorganisms.

In the Maritime Antarctic, densely vegetated sites are often related to bird activities [12]. Organic matter rich in carbon, nitrogen and phosphorus is added to the soil in the form of guano, feathers, eggshells and birds remains which leads to the formation of ornithogenic soils [4]. These soils can occur at active or abandoned penguin colonies, but also nearby rookeries of other large birds (e.g. skuas, petrels). Birds not only change soil conditions for microbial life, they also inoculate microorganisms through guano deposition, and the composition of this guano seems to be influenced by penguin diet. Several studies reported *Firmicutes* 16S rRNA gene sequences related to ornithogenic soils [4], [11], [13] and, accordingly, Zdanowski and colleagues [14] isolated *Gammaproteobacteria*, *Firmicutes*, *Actinobacteria*, and *Bacteroidetes* from Adelie penguin guano.

Recent studies have provided a baseline knowledge of the microbial communities in terrestrial Antarctic environments [4], [11], [13], [15]–[19]. For instance, the phylum-level diversity in Admiralty Bay rhizosphere soils was much higher than previously reported, with the phyla *Firmicutes* and *Actinobacteria* representing more than 70% of the total community [11]. In contrast, along a latitudinal transect in the Maritime Antarctic, bacterial communities were dominated by the phyla *Proteobacteria*, *Actinobacteria*, *Bacteroidetes*, *Acidobacteria* and *Cyanobacteria*, with a strong influence of the vegetation cover, highlighting the potential for indirect effects of global warming on Antarctic soils [19]. In a recent report, *Acidobacteria*, *Bacteroidetes*, *Proteobacteria* and *Firmicutes* were the main phyla recovered in nine different soils of Livingston Island, Maritime Antarctic [20]. In soils from the Ross Sea region, Continental Antarctic, the dominant bacteria were related to the *Acidobacteria*, *Actinobacteria*, *Bacteroidetes*, *Proteobacteria* and *Firmicutes* [3].

Global warming is currently affecting both plant [2] and bird distribution and activity in Antarctica [21] and can also influence the activity of the microbial communities [18] In view of the strong influence birds and vascular plants have on Antarctic soil microorganisms, it is crucial to understand this relationship in order to better predict any indirect effects global warming might have on soil microorganisms. In this paper we focus on the differences between community structure and composition at the phyla/class levels in *D. antarctica* and *C. quitensis* rhizosphere and in bulk soils from six different sampling sites around Admiralty Bay, King George Island, Maritime Antarctic. Soils with different levels of bird influence were compared to adjacent rhizosphere soils using 16S rRNA gene microarrays and phylum/class-specific quantitative PCR genes and the resulting patterns were analyzed together with soil physico-chemical parameters.

## Materials and Methods

### Ethics Statement

All necessary permits were obtained for the described field studies, from the Brazilian Antarctic Program, PROANTAR, as part of the IPY (International Polar Year) Activity # 403 and MMA (Brazilian Ministry of Environment).

### Sampling Site and DNA Extraction

The study was carried out at the Brazilian Antarctic Station Comandante Ferraz (EACF, 62°04′S, 58°21′W), located in Martel Inlet, Admiralty Bay, King George Island, Antarctic Peninsula, which is part of the South Shetlands Archipelago in Maritime Antarctica. During the austral summers of 2006–2007 and 2008–2009, the vascular plants D. antarctica or C. quitensis were sampled, where both plants were found, in triplicate at six different sites: A – Arctowski (E:423.807/N: 3.106.863–2006–2007), Q – Quimica (E: 427.335/N: 3.115.506–2006–2007), I – Ipanema (E: 426.570/N: 3.116.513–2006–2007), M – North Mountain (E: 426.63/N: 3.116.587- 2008–2009), D – Demay Point (E: 425.102/N: 3.100.854–2008–2009), C – Copacabana (E: 424.706/N: 3.105.149–2008–2009). Colobanthus quitensis were not found at points C and M. Points A, C and D were located inside an environmental protected area. Point A is close to the Arctowski Polish Station and next to a colony of Adelie penguins (Pygoscelis adeliae), point C is next to the USA summer station Copacabana in a Gentoo penguin (P. papua) colony, and point D is near to a Polish refuge next to a colony of Chinstrap penguins (P. antarcticus). At point I, there were no penguin colonies present, but this section was used as a nesting site by local species of flying birds. Point Q was located in the vicinity of the EACF; thus there has been (and continues to be) an intense anthropogenic influence on this spot, which is not the case at the other sampling sites. Besides, there is a transient presence of birds. Point M was located at the top of North Mountain, around 200 m altitude. This site has no influence from penguin colonies and only a few nests of skua (Catharacta sp.) were observed.

At each sampling site, triplicate soil samples (500 g of surface soil cores, 0–5 cm) were taken for chemical and biological analyses, with the exception of the Arctowski site (A) where we only take two replicates. Soil samples were preserved at 4°C for chemical and at −20°C for biological analyses. The plants occur in the same area, not necessarily growing one next to the other. Furthermore, we collected rhizosphere soil from isolated plant roots. Each vascular plant sample was frozen (−20°C) at the EACF. To assess the bacterial communities associated with the rhizospheres of both vascular plants, 5 g of the roots with aggregated soil was shaken in a 45 mL of saline solution (NaCl 0.85%) for 2 hours. Then, the supernatant (without roots) was centrifuged for 10 minutes at 9,000 rpm, and 0.5 g of the pelleted soil was used for DNA extraction using the Fast DNA Spin Kit for soil (QBIOgene, Carlsbad, CA) following the manufacturer’s instructions. For the bulk soil, 0.5 g of each sample was used for the extractions also using the Fast DNA Spin Kit. The extracted DNA was quantified using the PicoGreen® dsDNA quantitation assay (Invitrogen, Carlsbad, CA). The integrity of the DNA extracted from the soils was confirmed by electrophoresis on a 0.8% agarose gel in 0.5x TBE buffer (45 mM Tris–borate, 1 mM EDTA, pH 8.0).

### Soil Analysis

Chemical analyses on air dried, sieved (2 mm), subsamples of bulk soil samples were performed. A single subsample/replicate was used for the following analyses. Exchangeable cations: Ca^2+^, Mg^2+^ and Al^3+^ extracted in 1 M KCl; P, Na and K by Mehlich-1 extractant (HCl 0.05 mol L-1 and H2SO4 0.0125 mol L-1)and pH (soil: water, 1∶10); Potential acidity: H+Al extracted with 1 N calcium acetate (pH 7), titrated with 0.0125 NaOH N, were analysed according to [22]. Inductively coupled Plasma apparatus for Ca^2+^, Mg^2+^ and Al^3+^, flame emission (K and Na) and photocolometry (for P) were used for nutrient determinations. Total organic C was determined by wet combustion [23].

For the nitrogen quantification, all the bulk soils replicates were analyzed. Mineral nitrogen forms were first extracted from the soil samples with a KCl (2N) solution. The ammonium (NH_4_^+^) and nitrate (NO_3_^−^) contents in the extracts were determined by use of an automatic flow injection (FIA) analysis system. The ammonium (NH_4_^+^) was quantified colorimetrically using the Solorzano method [24], and the nitrate (NO_3_^−^) estimated by conductivimetry in the form of nitrite (NO_2_^−^), after being reduced with a cadmium base catalyst.

### Real-time Quantitative PCR (qPCR)

The primers and qPCR conditions used are summarized in Table 1. qPCR was performed in 20 µl volumes using the iQ ™ SYBR® Green Supermix Kit (Bio-Rad Laboratories, Carlsbad, CA) on a Rotor-Gene 3000 apparatus (Colbert Life Science, Sidney, NSW, Australia). Reactions were set up as per the manufacturer’s instructions, with 0.5 µM of each primer, 0.4 mg/ml of BSA (bovine serum albumin) and 1 ng of total soil DNA extract. The temperature profile included an initial hot start for 3 min at 95°C; and PCR cycling and detection (40 cycles) for 30s at 95°C, 30s at the stated annealing temperature (Table 1), and 30s at 72°C (acquiring signal at the end of this step). Standards were made from 10-fold dilutions of linearized plasmids containing the gene fragment of interest that was cloned from amplified soil DNA. For all reactions, no-template controls were carried out and yielded no detectable signals. Lambda DNA was used to correct for potential PCR inhibitors present in soil extracts [29]. Equal volumes of 10-fold diluted soil DNA extract and of a cloned 500-bp fragment of bacteriophage lambda (10^5^ copies per µl) were mixed. When the recovery of lambda was below 100%, quantification values for all other genes were corrected accordingly.

**Table 1:** Primers and annealing temperatures used in the Real Time PCR assays.

Target	Primers (For./Rev.)	Annealing temp. (°C)	Reference
Total Bacteria 16S	Eub338/Eub518	53	[25]
Acidobacteria	Acid31/Eub518	50	[25]
Actinobacteria	Actino235/Eub518	60	[25]
Alphaproteobacteria	Eub338/Alf685	60	[25]
Bacteroidetes	Cfb319/Eub518	65	[25]
Betaproteobacteria	Eub338/Bet680	60	[25]
Epsilonproteobacteria	Eps549/Eub338	56	[26] [25]
Firmicutes	Firm934/Firm1060	60	[27]
Gammaproteobacteria	Eub518/Gamma871	56	[25] [28]

### 16S rRNA Gene PCR Amplification and Microarray Analysis

For the microarray assays all the triplicate rhizosphere and bulk soil samples from the six sample sites were used. A 16S rRNA gene fragment was amplified using the universal primers F1-R13 [30]. The PCR mixture comprised 1 µl of template DNA (10–20 ng), 20 pmol of universal primers, 5 µl of 10×PCR buffer (Fermentas, Burlington, Ontario, CA), 1.5 mM MgCl_2_, 2.5 U of Taq DNA polymerase (Fermentas), 0.2 mM of each dNTP and sterile filtered water to a final volume of 50 µl. The temperature profile included an initial denaturation step at 94°C for 5 min, 35 cycles of a denaturation step at 94°C for 30s, a primer annealing step at 50°C for 30s and an extension step at 72°C for 45s, followed by a final step of 7 min at 72°C. PCR products were purified using the PureLink ™ PCR purification Kit (Invitrogen Canada, Burlington, Ontario, CA). A microarray platform targeting the 16S rRNA genes of Bacteria and Archaea found in cold environments was used as previously described [31]. To summarize, the purified 16S rRNA genes were chemically labeled with Cy5 and hybridized overnight onto the microarray. Details about the microarray and probe design are available at the National Center for Biotechnology Information (NCBI) GEO database under the platform accession number GPL8953. The presence-absence of the different Bacteria and Archaea targeted by the microarray was used as community profile to evaluate the similarity between the bulk soil and rhizosphere samples from the six different sampling sites. Microarray data discussed here have been deposited in NCBI GEO and are accessible through GEO series accession no. GSE33847.

### Statistical Analyses

All statistical analyses were performed in R (version 2.9.0, The R Foundation for Statistical Computing). For analysis of variance (ANOVA) of the qPCR data, normality was tested using the “shapiro.test” function and homoscedasticity was tested using the “bartlett.test” function. When necessary, data were log transformed to meet parametric ANOVA assumptions. ANOVA and post hoc Tukey HSD tests were then carried out using the “aov” and “TukeyHSD” functions, respectively. When transformations failed to normalize data, Kruskal-Wallis and associated multiple comparison tests were performed using “kruskal.test” and the “kruskalmc” functions of the “pgirmess” library, respectively. Spearman linear correlation analyses (correlations on qPCR vs. soil data) were performed using the “cor.test” function. For the microarray analysis, distance matrices were calculated using the ”vegdist” function of the ”vegan” library and principal coordinate analyses (PCoA) were carried out using the ”cmdscale” function based on the square root of 1-Jaccard. PerMANOVA [32] of microarray data was carried out on Jaccard similarity, testing separately for the influence of plants (*C. quitensis*, *D. antarctica*, no plant), site and nitrogen on the microbial community structure. Spearman linear correlation analyses (correlations on qPCR vs. soil data) were performed using the “cor.test” function. Levels of significance were corrected for multiple comparisons using the method of Bonferroni [33].

## Results

### Soil Characteristics

The three sites located in penguin colonies (A, C and D) had similar soil chemical characteristics, with low pH and high bioavailability of P (Table 2). The two others sites under the influence of birds (Q and I) also had high values of organic C and P, but only a moderate pH. The M site, which was not directly under the influence of birds, had the highest pH value (8.2), exchangeable Ca and S, but the lowest value for P. The nitrogen concentrations (NH_4_^+^ and NO_3_^−^) were very high for the sites under the influence of penguins, very low in the sites under the influence of other birds (Q and I) and below the detection limit for the M site. For all sampling sites NH_4_^+^ values were higher than NO_3_^−^ values. Site C had nitrogen values similar to the other sites under the influence of penguins, but lower values of exchangeable Ca and Mg.

**Table 2:** Mean soil characteristics for surface soil cores (0–5 cm) collected in six sampling sites at Admiralty Bay, King George Island.

Sample sites	Na	Ca	Mg	K	H+Al	Al	S	pHwater	Corg	P	K	N-NH_4_	N-NO_3_
	Cmol_c_ dm^−3^							1∶2.5	g kg^−1^	mg l^−1^		µg g^−1^	
Arctowski	0.49	12	7	0.02	20	3.55	19.51	4.3	0.97	1005	9	590.11	13.98
Ipanema	0.74	11	6	0.02	11	0	17.76	5.7	5.11	786	9	2.31	0.051
Química	0.31	9.4	5.6	0.32	1.3	0	15.63	6.6	2.28	620	128	7.21	0.891
Demay Point	0.32	5.3	8.3	1.3	22.6	14.9	15	4.9	5.3	464.2	429	263.91	10.51
North Mountain	0.25	28.5	4.5	0.12	0	0.9	33.4	8.2	1.9	349	47	n.d.^#^	n.d.
Copacabana	0.47	4.2	1.3	0.64	7.4	0.9	6.6	4.8	1.2	649.1	250	361.56	14.07

# n.d.: not detected.

### Rhizosphere vs. Bulk Soil

**Figure 1:**
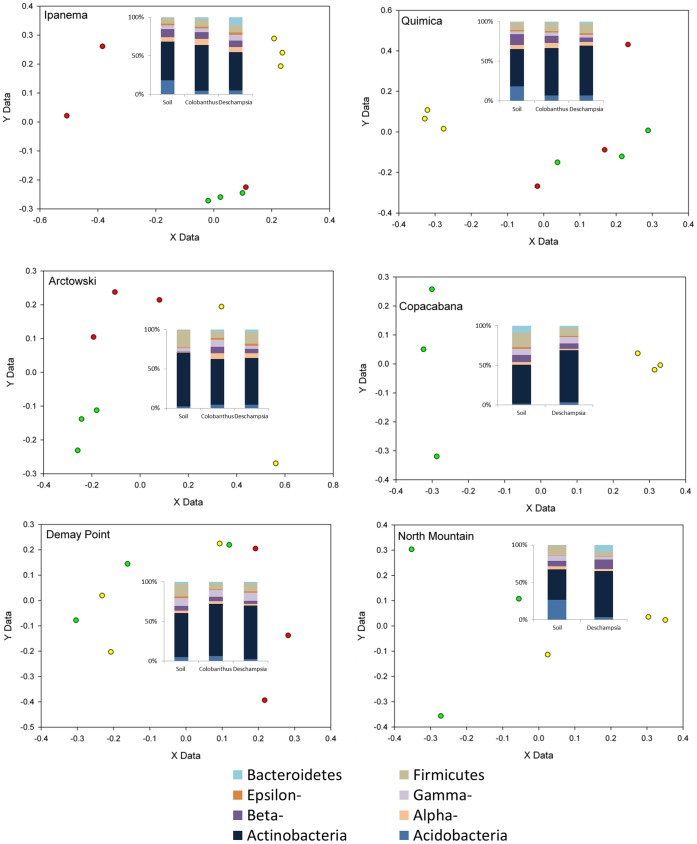
Rhizosphere effect on microbial community structure and relative abundance. Principal coordinate analysis calculated based on the microarray results for each sampling site separately. Only presence or absence of the probes was considered. All the replicates are represented in the figures. Red dots represent *Colobanthus quitensis;* Green dots represent *Deschampsia antarctica*; and yellow dots represent bulk soil samples. *Insets*: relative abundance of the main bacterial phyla quantified by qPCR. An average of the qPCR triplicates results of each sample is presented.

A microarray targeting the 16S rRNA genes of microorganisms frequently found in cold environments consisting of 525 25-mer oligonucleotides targeting 159 bacterial and archaeal taxa was used to compare bulk soil and rhizosphere samples. Out of these 159 taxa, 13 to 41 taxa were detected per sample. Principal coordinate analyses of the microarray results for bulk soil and rhizosphere samples were made separately for each sampling site (Fig. 1). Figure 1 shows a clear separation of the bacterial communities from bulk soil and rhizosphere at most sites with the exception of the D and M sites. This visual interpretation was confirmed by PermANOVA analyses that tested for significant differences between bulk soil and rhizosphere samples at each sampling site separately (Table 3). No clear differences between the microbial communities of the rhizosphere of *C. quitensis* and *D. antarctica* were detected for most sampling sites, with the exception of the A site where it was possible to see a clear segregation between the microbial communities associated with the rhizosphere of the two plant species (Fig. 1).

**Table 3:** Test for significant difference between rhizosphere soils and bulk soil using PermANOVA for all the different sites (microarray data).

	Arctowski	Copacabana	DemayPoint	Ipanema	N. Mountain	Quimica
F	**0.6335**	**0.7203**	0.3024	**0.4809**	0.411	**0.4224**
P	**1.00E-04**	**0.0465**	0.2129	**0.0095**	0.0779	**0.0325**

Significant values (P<0.05) are in boldface.

**Figure 2:**
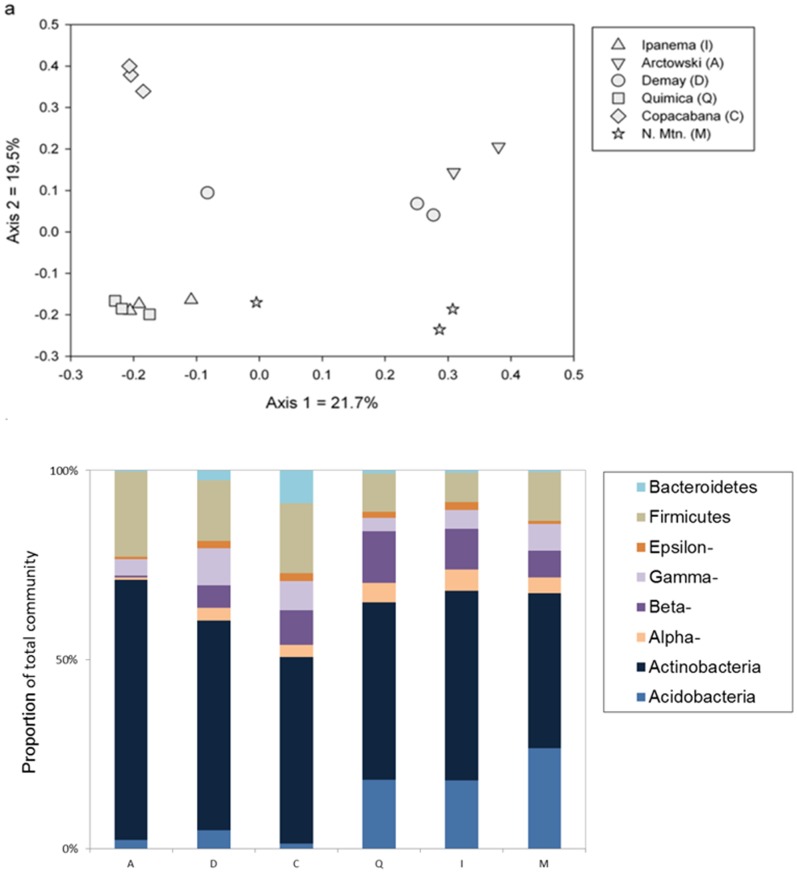
Differences in microbial community structure and relative abundance in bulk soil between sampling sites. a) Principal coordinate analysis based on the microarray results. Only presence or absence of the probes was considered. Here only bulk soil samples were analyzed. All the bulk soil replicates are represented in the figures. b) Relative abundance of the main bacterial phyla quantified by qPCR assays. An average of the qPCR triplicates results of each sample was made.

The microbial communities of different sites around Admiralty Bay were compared (Figs 2a and 2b). According to the qPCR results, *Actinobacteria* was the most abundant phylum, followed by the *Proteobacteria* (*Epsilon*-, *Gamma*-, *Beta*- and *Alpha*-) and the *Firmicutes* when considering all samples together. In the bulk soils from sites I, Q and M, *Acidobacteria* was also abundant (Fig. 2b). ANOVA analyses of the qPCR results were also made in order to compare rhizosphere and bulk soil samples at each of the six sampling sites separately (Table 4). For each sampling site at least one phylum showed significantly different abundances between bulk soil and rhizosphere samples. For instance, the *Firmicutes* were significantly more abundant in the bulk soil samples than in the rhizosphere samples for the A, C, D and M sites (Fig. 1, Table 4). Similarly, the *Acidobacteria* were significantly more abundant in the bulk soil samples than in the rhizosphere samples for the Q, I and M sampling sites (Fig. 1, Table 4). The *Actinobacteria* showed an opposite trend, being significantly more abundant in the rhizosphere samples for the Q and M sites (Fig. 1, Table 4).

**Table 4:** Anova analysis to test the differences in the phyla abundance (qPCR) between bulk soils and rhizospheres from the same sampling site.

Quimica								
Groups	Gamma	Firm	Alpha	Acido	Actino	Bacteroi	Beta	Epsilon
P-value	0.718	0.075	0.504	0.002	0.013	0.288	0.064	0.216
Soil	a	a	a	b	b	a	a	a
*Colobanthus*	a	a	a	a	a	a	a	a
*Deschampsia*	a	a	a	a	a	a	a	a
Demay								
Groups	Gamma	Firm	Alpha	Acido	Actino	Bacteroi	Beta	Epsilon
P-value	0.803	**0.035**	0.101	0.070	0.242	0.945	0.750	0.398
Soil	a	b	a	a	a	a	a	a
*Colobanthus*	a	a	a	a	a	a	a	a
*Deschampsia*	a	ab	a	a	a	a	a	a
Copacabana								
Groups	Gamma	Firm	Alpha	Acido	Actino	Bacteroi	Beta	Epsilon
P-value	0.510	**0.014**	0.100	**0.011**	0.099	**0.001**	0.567	**0.022**
Soil	a	a	a	a	a	a	a	a
*Deschampsia*	a	b	a	b	a	b	a	b
N. Mountain								
Groups	Gamma	Firm	Alpha	Acido	Actino	Bacteroi	Beta	Epsilon
P-value	**0.0007**	**0.002**	0.523	**0.0004**	**0.003**	0.203	0.179	0.373
Soil	a	a	a	a	a	a	a	a
*Deschampsia*	b	b	a	b	b	a	a	a
Ipanema								
Groups	Gamma	Firm	Alpha	Acido	Actino	Bacteroi	Beta	Epsilon
P-value	0.151	0.716	0.723	**0.0007**	0.117	**0.013**	0.660	0.428
Soil	a	a	a	b	a	b	a	a
*Colobanthus*	a	a	a	a	a	ab	a	a
*Deschampsia*	a	a	a	a	a	a	a	a
Arctowski								
Groups	Gamma	Firm	Alpha	Acido	Actino	Bacteroi	Beta	Epsilon
P-value	0.235	**0.005**	**0.012**	0.223	**0.003**	0.081	**0.001**	0.181
Soil	a	b	b	a	b	a	b	a
*Colobanthus*	a	a	a	a	a	a	a	a
*Deschampsia*	a	ab	a	a	a	a	a	a

Different letters within a column represent significant difference between samples. Significant values (P<0.05) are in boldface.

### Differences between Sites

A PCoA analysis was also carried out using only the microarray profiles of bulk soils in order to compare the general microbial community at different sites around Admiralty Bay (Fig. 2a). Replicate samples from a site clustered together in most cases, and differences were seen between most of the sites. PermANOVA analyses confirmed that there were significant differences between the microbial community structures of different sites (F = 6.87, P = 0.0001). Samples from sites A and D grouped together (with the exception of one site D replicate), while samples from sites Q and I also grouped together. Samples from sites C and M were unusual and did not clearly group with any samples from the other sites. According to ANOVA analyses (Table 5), *Acidobacteria* abundance was significantly higher at the M site and significantly lower at the C site. In contrast, the M site showed significantly lower abundance of *Actinobacteria* compared to all other sites (Fig. 2b). The *Bacteroidetes* phyla were significantly more abundant at the C site compared to most other sites. The *Alphaproteobacteria* were significantly more abundant at the I site And the *Gammaproteobacteria* were significantly less abundant in the Q site compared to the C and D sites. *Epsilonproteobacteria* had no difference among the bulk soils samples. *Betaproteobacteria* seems to vary among samples, especially comparing sampling points A and M to the other sampling points (D, C, Q and I), where this group were more representative.

**Table 5:** Anova analysis to test the differences in the phyla abundance between different bulk soils from the six sampling site in Admiralty Bay.

Bulk soil								
Groups	Gamma	Firm	Alpha	Acido	Actino	Bacteroi	Beta	Epsilon
P-value	0.002	0.0001	0.002	0.000002	0.00001	0.00007	0.027	0.1297
Copacabana	a	a	a	a	a	a	a	a
Demay Point	a	b	ab	b	a	ab	a	a
Ipanema	ab	b	ab	ab	a	b	a	a
N. Mountain	ab	ab	ab	c	b	b	a	a
Quimica	b	ab	ab	b	a	b	a	a
Arctowski	ab	b	b	ab	a	b	a	a

Different letters within a column represent significant difference between samples. Significant values (P<0.05) are in boldface.

**Figure 3:**
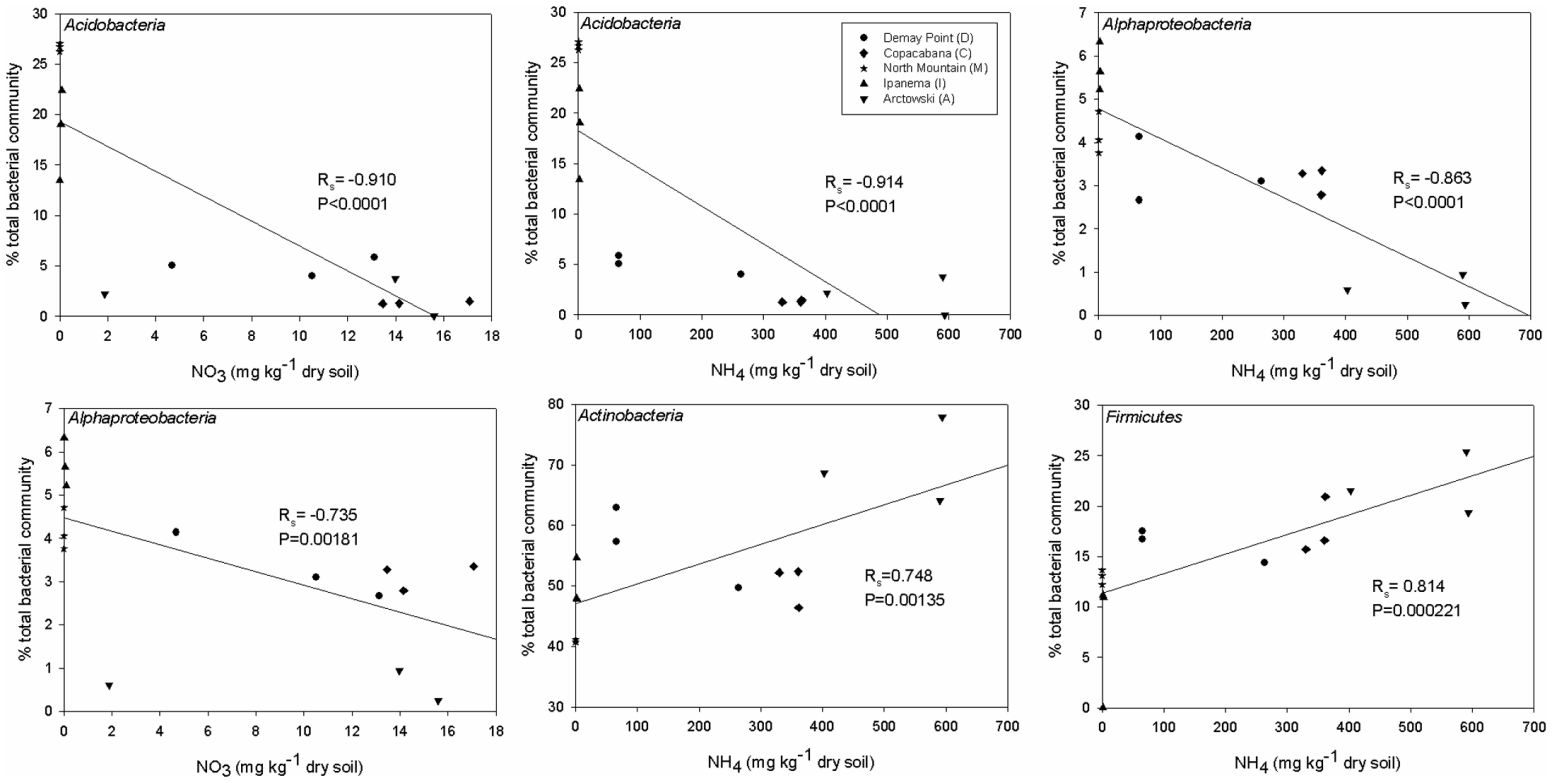
Correlation between phylum abundance (qPCR) and nitrogen quantification. Spearman coefficients (R) are shown for each taxon, with the associated Bonferroni-corrected P values.

The statistical correlations between the soil physical and chemical composition and measured microbial communities were performed, but gave no significant result for most soil abiotic characteristics (data not shown). Only NH4 and NO3 presented some correlation. In order to check the relationship between nitrogen and soil bacterial communities in Admiralty Bay, correlation analyses were carried out (Fig. 3). Using the Spearman coefficient, we found a significantly positive correlation between the *Actinobacteria* and the *Firmicutes* and the ammonia concentrations in soil (Fig. 3). A significantly negative correlation was found between the *Acidobacteria* and the *Alphaproteobacteria* and ammonia concentration. *Acidobacteria* and *Alphaproteobacteria* were also significantly and negatively correlated with nitrate concentration. For all the other bacterial groups-nitrogen concentration combinations, no significant correlations were found. We also carried out PermANOVA analyses on the microarray dataset using NH_4_^+^ and NO_3_^−^ as explanatory variables and found that the microbial community structure in bulk soil was significantly influenced by NH_4_^+^ (F = 4.04, P = 0.0009) and NO_3_^−^ (F = 3.30, P = 0.0064).

## Discussion

Our results showed that both plant and bird presence had significant influences on microbial community structure and on the relative abundance of several major bacterial taxa. The presence of birds was related to higher soil nitrogen concentrations, which was identified as significantly correlated with the abundance of several bacterial groups and as significantly influencing the microbial community structure. In the case of plants, it appeared that the effect observed was a general rhizosphere effect, as very little difference was seen between the rhizosphere of the two vascular plants sampled, in line with previous reports [11].

### Influence of Birds

As expected, we observed a strong relationship between microbial communities and soil characteristics, but we could not disentangle the effect of nutrient availability from any pH-related effect, as the sites with high nitrogen and phosphorus concentrations had also the lowest pH values. These characteristics are typical of ornithogenic soils as bird guano, feathers, eggshells and remains are rich in nitrogen and phosphorus [4]. In the Maritime Antarctic, densely vegetated sites covered by lichens, mosses and vascular plants are often related to bird activity [12]. The influence of birds in the development of Antarctic soils can occur at different levels. According to Simas and colleagues [34] the presence of flying birds, such as skuas (*Catharacta* sp), giant-petrels (*Macronectes* sp) and seagulls (*Larus* sp), leads to the development of weakly ornithogenic soils (moderately high N and P concentrations, slightly acidic pH) while the presence of penguin colonies leads to highly ornithogenic soils (very high N and P concentrations and acidic pH). Based on the results from our soil analyses and from previous studies of Admiralty Bay soils [12], [34], [35], we can roughly classified the soils from our sampling sites as follows: soils from sites A, C and D would be highly ornithogenic soils, soils from sites I and Q would be weakly ornithogenic soils and soil from site M would not be ornithogenic soil. According to [12], which used a comprehensive sampling, the amount of sand, silt and clay in ornithogenic soils are about 73±10, 15±7 and 11±4%, respectively, and 41±18, 32±4 and 21±8% to weakly ornithogenic soils. Soil from the M site, with high pH, high concentration of exchangeable Ca and low concentrations of organic C and total nitrogen could be classified as basaltic/andesitic soil according to [12], and presents about 38±16, 32±7 and 25±5% of sand, silt and clay respectively. The high S concentration found for this site could be attributed to the sulfide bearing andesides and related tills present in the region [12].

According to PCoA generated from the microarray data, the bacterial communities were strongly shaped by the nitrogen level in the soil, and consequently, to the bird influence. Samples of weakly ornithogenic soils generally grouped together (sites Q and I), while the non-ornithogenic soils (site M) formed a separate group. However, for the highly ornithogenic soils (sites A, D and C) two separate groups could be observed: one comprising samples from sites D and A, and another comprising only samples from site C. Interestingly, each of these sites is occupied by different penguin species which have different diets. Studies on penguin diet showed that *Pygoscelis adeliae* and *P. antarctica* (the species found in sites A and D, respectively) feed on krill, mainly *Euphrasia superba*, while *P. papua* (the species found in site C) eats krill but also fish, particularly *Notothenia* species [36]–[38]. The diet of the penguin could influence soil microbial communities in two ways: through modification of the penguin’s gut microbiota and thus in the microbial inputs to soil through guano deposition [4], and through modification of the physico-chemical composition (N, P and pH) of the penguin’s guano. We hypothesize that the diet of *P. papua*, which colonizes site C, is at least partially responsible for the different microbial community patterns observed at this site.

Corroborating the idea that bird presence has a fundamental role in shaping the microbial communities of Maritime Antarctic soils, due to the high N and P inputs in the soils, samples from the non-ornithogenic M site presented the most distinctive bacterial community structure and composition, probably due to the low availability of nutrients and higher pH. The *Acidobacteria* were more abundant in the non- and weakly ornithogenic soils and were consequently negatively correlated to ammonia. Similarly, Ganzert and colleagues [39] reported *Acidobacteria* sequences were related to Antarctic soils with low C and N contents and neutral to alkaline pH values. In contrast, the *Firmicutes* and the *Actinobacteria* were positively related to ornithogenic soils, in line with previous studies [4], [11], [13]. Furthermore, Zdanowski and colleagues [14] isolated *Gammaproteobacteria*, *Firmicutes*, *Actinobacteria*, and *Bacteroidetes* directly from Adelie penguin guano. From our data it is not possible to deduce any functional information about the microbial communities studied, but Zhu and colleagues [40] reported that the ammonia concentration in the penguin guano is much higher than the nitrate concentration, which could favor nitrifying bacteria in ornithogenic soils. However, further studies focusing on the N-cycle using a functional approach will be needed to confirm this hypothesis.

### Influence of Plants

It was recently hypothesized that microbial communities were mainly driven by soil characteristics, but that within a soil type, plants shaped the microbial communities in their rhizosphere [41]. In view of the very large differences between the soils characteristics of the different sampling sites used in this study, we limited our search for a rhizosphere effect to each site separately. Even though the microbial communities were predominantly driven by differences in soil characteristics, microbial communities within a site were significantly influenced by the presence of plants. Within each individual site, we found significant differences between the microbial communities found in bulk soil and rhizosphere samples in both the microarray and the qPCR datasets. Similarly, vegetation cover was previously reported as having a strong influence on the bacterial community composition in a range of maritime Antarctic sites [19]. In more temperate settings, it was also previously reported that bulk and rhizosphere soils harbored different microbial communities [5], [42]–[45]. Plants select for a specific microbial community in their rhizosphere through root exudation [46] and since different plants exude different compounds, the rhizosphere effect is thought to be plant-specific [43]. However, in the present study, we did not find any clear difference between the microbial communities in the rhizosphere of *C. quitensis* and *D. antarctica*, in line with a previous report that used pyrosequencing and DGGE [11]. Other previous studies also report similar results, suggesting that the soil characteristics could be a determinant factor shaping the microbial communities instead of the rhizosphere plant species [9], [10].

In a previous study from our group that used 16S rRNA gene pyrosequencing on *D. antarctica* and *C. quitensis* rhizosphere samples from Admiralty Bay, it was shown that the *Firmicutes* were the most abundant phylum followed by the *Proteobacteria* and then the *Actinobacteria* [11]. In the present study, although the same three groups were still the most abundant, the qPCR approach showed a dominance of *Actinobacteria* followed by *Proteobacteria* and *Firmicutes*. These discrepancies could be due to the fact that we did not use primers targeting the same positions in the 16S rRNA gene for both techniques. Furthermore, the primers used for the *Actinobacteria* in qPCR are not perfectly specific to this group and unspecific amplifications could artificially increase their abundance in qPCR assays [47]. However, the main goal of this study was to compare bulk soils and rhizospheres from different sampling sites and the results from these comparisons are valid since all samples were subjected to the same bias. Our results nonetheless showed that the *Actinobacteria* and the *Proteobacteria* are the main bacterial taxa present in the soils of Admiralty Bay. Similar results were found in several other studies on soil bacterial diversity around the world [11], [48], [49] and in maritime Antarctica [11], [16], [27].

### Conclusions

Our results showed that the presence of vascular plants (*D. antarctica* and *C. quitensis*) and of birds play important roles in shaping the microbial communities of Antarctic soils. For the plant influence, the observed rhizosphere effect varied between the different sampling sites, probably due to differences in soil parameters, indicating that plant colonization will not have the same effect on microbial communities in all soils. Firmicutes and Acidobacteria were more abundant in bulk soils while Actinobacteria was more abundant in rhizosphere samples. For the bird influence, the effect varied with the level and the type of ornithogenic input and, in highly ornithogenic soils, seemed to be also modulated by the species of bird present, due to differences in diet. This indicates that the soils associated with bird colonies will influence microbial communities differently depending not only on the type of birds (flying birds vs. penguins), but also on the diet of the birds. Based on these results, we can hypothesize that not only will climate change directly influence Antarctic soil microbial communities, but it will also indirectly influence them through changes in plant and bird populations.

Further research will be conducted to directly test the guano (chemical and microbiological) of different penguins and even test the chemical attributes of exudates (or rhizosphere) of the two plants to confirm/deny the correlations, which may explain the resulting overlapping bacterial communities.
